# Perceptions of HPV and HPV vaccines among parents and caregivers of girls aged 9–14 years in Nigeria: a qualitative study

**DOI:** 10.1186/s12982-025-01276-0

**Published:** 2025-12-21

**Authors:** Stephen Olabode Asaolu, Daradara Kubura, Grace Erekosima, Mahfus Dauda, Grace Popoola, Rauf Rauf, Morenike Oni, Olugbemisola Samuel, Stallone Ngobua, Yusuf Musa, Al-Mustapha Mukhtar, Sunday Atobatele, Sidney Sampson, Hilary Okagbue

**Affiliations:** 1 Sydani Institute for Research and Innovation, Sydani Group, Abuja, Nigeria; 2 Sydani Initiative for International Development, Sydani Group, Abuja, Nigeria; 3 Sydani Group, Abuja, Nigeria

**Keywords:** HPV, HPV vaccine, Parents, Caregivers, Perceptions, Nigeria, Cervical cancer, Qualitative study

## Abstract

**Background:**

Human papillomavirus (HPV) vaccination is a critical strategy for preventing cervical cancer. Understanding community perceptions is essential to improving uptake, especially in low- and middle-income countries like Nigeria, where vaccine coverage remains suboptimal.

**Objective:**

To explore the perceptions of parents and caregivers regarding HPV and the HPV vaccine among girls aged 9–14 years in Nigeria.

**Methods:**

A qualitative study was conducted using in-depth interviews with 72 parents, caregivers, and school administrators across 9 selected sites in 5 geopolitical zones of Nigeria. Participants were recruited through purposive sampling. Interviews were audio-recorded, transcribed, and analyzed thematically using the Nvivo software version 14.

**Results:**

Participants demonstrated a general awareness that the HPV vaccine was freely available through government-supported programs. Vaccine acceptance was motivated by the desire to prevent cervical cancer and protect children’s long-term health. Parents and school administrators perceived HPV infection as a serious health threat with implications for education, economic stability, and future well-being. However, misconceptions about transmission routes and perceived susceptibility were common. School administrators recognized the relevance of the vaccine and viewed themselves as key advocates for its uptake.

**Conclusion:**

While there is strong support for the HPV vaccine among parents and educators, persistent knowledge gaps and sociocultural beliefs may hinder broader acceptance. Strengthening health education and leveraging schools as vaccination hubs may improve uptake and sustainability of HPV immunization programs in Nigeria.

**Supplementary Information:**

The online version contains supplementary material available at 10.1186/s12982-025-01276-0.

## Background

Human papillomavirus (HPV) is a common sexually transmitted DNA virus, and a subset of high-risk types (notably HPV-16 and HPV-18) are the causal agents for almost all cervical cancers and a substantial proportion of other anogenital and oropharyngeal cancers [1]. Globally, cervical cancer — overwhelmingly attributable to HPV infection — remained the fourth most common cancer in women in 2022, with roughly 660,000 new cases and 350,000 deaths recorded that year, the vast majority occurring in low- and middle-income countries [2, 3]. Sub-Saharan Africa bears a disproportionate share of this burden: the region accounts for a large fraction of global cervical cancer incidence and mortality, reflecting gaps in HPV vaccination, screening and treatment services [4, 5]. In Nigeria, estimates indicate about 12,000–15,000 new cervical cancer cases and roughly 8,000–10,000 deaths annually, making cervical cancer one of the leading causes of cancer morbidity and mortality among women in the country [6, 7].

The introduction of the human papillomavirus (HPV) vaccine into Nigeria’s routine immunization schedule in October 2023 marked a significant milestone in the country’s public health efforts [8–10]. This initiative aimed to vaccinate approximately 7.7 million girls aged 9–14 years, representing the largest single-round HPV vaccination campaign in the African region [9, 11]. Despite this ambitious rollout, challenges persist in achieving optimal vaccine uptake, particularly concerning the perceptions and attitudes of important stakeholders who are pivotal in the decision-making process for adolescent health interventions [12].

The free vaccine rollout for the young girls in Nigeria employed three strategies; community fixed posts, mobile outreaches, and school-based outreaches [13]. Notwithstanding the extant availability of the vaccine, its uptake is influenced significantly by the perceptions and attitudes of parents, caregivers and school administrators, who are primary decision-makers for the health of girls in the target age group. Before the roll out of the free vaccine, several barriers impede optimal vaccine uptake in Nigeria. These include financial constraints, limited access to healthcare facilities, inadequate knowledge about the vaccine, fears related to potential side effects, and cultural or religious beliefs [14–16]. Parental awareness and acceptance of the HPV vaccine are critical determinants of its successful implementation. Studies conducted in various regions of Nigeria have highlighted a general lack of knowledge about HPV and its associated health risks among parents and caregivers [17–21]. A study in Kano State revealed that only 4.2% of parents had heard of HPV, and a mere 5.1% were aware of the HPV vaccine [20]. Another study conducted in Ogun State revealed that while 82.4% of parents and caregivers had good knowledge of the HPV vaccine, only 47.7% were well-informed about cervical cancer [21]. Notably, many respondents became aware of the vaccine during the introduction campaign, highlighting the impact of targeted awareness programs [21]. This limited awareness contributes to vaccine hesitancy, with concerns about vaccine safety, potential side effects, and misconceptions about the vaccine promoting promiscuity being prevalent [22]. Addressing these perceptions using culturally sensitive education campaigns is essential to enhance vaccine acceptance and coverage [17, 23].

The role of healthcare providers is pivotal in shaping vaccine acceptance and uptake, particularly for newer vaccines like the HPV vaccine. Studies consistently show that recommendations from trusted health professionals are among the strongest predictors of vaccine acceptance among parents and caregivers [24]. In Delta State, healthcare workers were identified as the primary source of information about the HPV vaccine for 60.7% of mothers [25]. Healthcare providers not only serve as sources of credible information but also help to dispel myths and address concerns about vaccine safety, efficacy, and necessity. In low- and middle-income countries, including Nigeria, where misinformation and cultural misconceptions about HPV and cervical cancer persist, the proactive engagement of healthcare workers is even more critical [26]. A strong provider endorsement can bridge the gap between awareness and action, especially in communities with limited access to health education. Moreover, when healthcare providers receive adequate training and institutional support, they are more likely to initiate vaccine-related conversations and facilitate informed decision-making among parents [27]. Therefore, strengthening the capacity of frontline health workers through continuous education and communication skills training remains an essential strategy for improving HPV vaccine uptake [28]. The integration of school-based vaccination programs has shown promise in increasing coverage [29]. Engaging educational institutions not only facilitates access to the target demography but also provides an avenue for disseminating accurate information to both students and parents.

After the phase I of the HPV vaccine introduction in Nigeria, the Nigerian government through the National Primary Healthcare Development Agency (NPHCDA), State Primary Healthcare Development Agencies (SPHCDA) and Development Partners launched the phase II of the vaccine introduction between May and June, 2024. The phase II was successful in some states, while some local government areas (LGAs) had lower performance in terms of vaccine acceptance. Given these considerations, this study aims to explore the perceptions of parents, caregivers and school administrators regarding HPV and HPV vaccine for girls aged 9–14 in Nigeria who have received the vaccine. Understanding these perceptions is vital for developing effective strategies to enhance vaccine acceptance and coverage in future vaccine introductions, ultimately contributing to the reduction of cervical cancer incidence in the country.

## Methodology

### Study design

This study adopted a qualitative research approach to explore the perceptions of HPV and HPV vaccine among parents and caregivers of girls aged 9–14 years in Nigeria. Qualitative approach was chosen for its ability to elicit rich, descriptive data and facilitate an in-depth understanding of the experiences, practices, and insights of stakeholders involved during the vaccine introduction.

### Study setting

The study was conducted in the nine states selected from the 21 human papillomavirus vaccine introduction (HPVVI) Phase II states where the Sydani Group had been contracted to provide technical support. These states were reported to represent diverse geopolitical zones of Nigeria, ensuring a comprehensive and representative scope for the research. The states included Cross River, Edo, and Delta from the South-South region; Kogi, Kwara, and Plateau from the North-Central region; and Ekiti, Ebonyi, and Katsina, representing the South-West, South-East, and North-West regions, respectively. The selection of these states aimed to capture regional diversity in sociocultural, economic, and health system contexts. This diversity was highlighted as critical for understanding the unique and common factors influencing HPV vaccine perception and uptake across different settings. In each state, two local government areas (LGAs) were further selected; the highest performing LGA and the lowest performing LGA, except in Kwara state where the lowest performing LGA was security compromised and inaccessible and the second lowest reporting LGA was selected to replace (Table 1).

**Table 1:** Local government areas selected in the nine States

State	Highest performing LGA	Lowest performing LGA
Cross river	Bakassi	Calabar Municipal
Ebonyi	Ebonyi	Ezza North
Edo	Oredo	Ovia North-East
Ekiti	Ido-Osi	Ekiti South-West
Delta	Ika North-East	Oshimili South
Katsina	Katsina	Kurfi
Kogi	Ogori Mangogo	Okene
Kwara	Offa	Ifelodun
Plateau	Jos South	Barkin Ladi

### Sampling size and strategy

The study employed a purposive sampling method to select respondents for the interviews. This non-probability sampling technique was chosen to ensure that participants were specifically selected based on their ability to provide rich, relevant, and detailed information regarding the research objectives. Parents of eligible children who accepted the vaccine and school administrators of schools were the school-based campaign was conducted were interviewed. Two parents and two school administrators were interviewed in each LGA, giving rise to eight in-depth interviews (IDIs) per state and 72 IDIs in total. Parents whose daughters did not receive the vaccine and school administrators whose school did not participate in the vaccine campaign were excluded from the study.

### Study guide development

Structured In-Depth Interview (IDI) guides tailored to the key participant groups were employed as primary data collection tools. The interview guide for parents has three sections: Section A – Socio-demographics, Section B: Awareness of HPV and the HPV vaccine, and Section C - Perceived susceptibility, severity and benefits of the vaccine. Similarly, the guide for the school administrators also has three sections: Section A – Socio-demographics, Section B: Knowledge and awareness of HPV and the vaccine, and Section C: Perceived factors that influenced the vaccine introduction and uptake (supplementary material 1 & supplementary material 2). The interview guides were developed using the health belief model [30]. They were pretested and piloted with a small group in Federal Capital Territory (FCT), similar to the target respondents to ensure the flow of the questions and prompts, and feedback from the exercise was incorporated into the guides.

### Data collection

The data collection was conducted by a team of research assistants who reside in the implementation sites. Their activities were supervised by the research team from the Sydani Institute for Research and Innovation (SIRI) to ensure the reliability and validity of the collected data. The data collection took place over a two-week period in October, 2024. The interviews were conducted in English and the local languages depending on the respondents’ language preference. Respondents were interviewed at their offices, homes or at other locations they suggested as convenient. Each interview session lasted between 25 and 45 min. The interview sections were recorded using an audio recorder and the audio files were anonymized and uploaded to a secure system immediately after each interview. The supervision by the SIRI research team ensured that research protocols were followed, the interview process remained consistent, and the quality of the data met the required standards. This approach facilitated the effective and efficient collection of qualitative data in alignment with the study’s objectives.

### Managing risk of bias

Given that this study was conducted in a location where Sydani Group provides technical support, we recognized the potential risk of bias in social desirability or perceived obligation by participants to respond favorably. To mitigate this, data collection was conducted by independent research assistants who were not affiliated with the technical support team and who clearly communicated the independence of the research from any service delivery or programmatic evaluation activities. Additionally, participants were assured of confidentiality, and interviews were conducted in neutral, non-threatening environments to foster openness.

### Data analysis

The responses obtained from the in-depth interviews were transcribed, translated (where applicable), and analyzed thematically. The process involved identifying themes, sub-themes, and re-emerging themes using the NVivo software, which is specifically designed for qualitative data analysis. Thematic coding was applied to organize the data into categories that aligned with the research objectives. Relevant quotes from the coded transcripts were extracted and used to illustrate key findings, providing direct insights into participants’ perspectives as they related to the research questions.

### Ethical considerations, approval, and consent

This study was conducted in accordance with the Helsinki Declaration. The study protocol was reviewed by research ethics experts at the Sydani Institute for Research and Innovation (SIRI) and approved by the National Health Research Ethics Committee (NHREC) with approval number NHREC/01/01/2007-31/10/2024. Confidentiality and data protection measures were upheld. Data were anonymized and stored securely in password-protected systems accessible only to authorized personnel. Participants were informed about the study’s purpose, potential risks, and benefits, and willingly gave their consent. Following global research ethics standards, all data generated from the study were stored electronically and will be retained for a minimum of five years to ensure proper management and potential future reference.

## Results

### Socio-demographic description of participants

The study included 72 participants, the vast majority of whom were female (81.9%). The age distribution was varied, with the largest group of participants being between 50 and 59 years old (33.3%). In terms of education, the group was highly qualified; a combined 62.5% of participants had attained either tertiary (51.4%) or postgraduate (11.1%) degrees. Civil servants constituted the primary occupation among the parents, representing 61.1% of the sample. Among the parents surveyed, most (69.4%) had one eligible daughter (Table 2).

**Table 2:** Socio-demographic characteristics of participants (*n* = 72)

Characteristic		Frequency (*n*)	Percentage (%)
Age	< 30 30–39 40–49 50–59 ≥ 60	5 21 19 24 3	6.9 29.2 26.4 33.3 4.2
Gender	Male	13	18.1
	Female	59	81.9
Level of Education	Primary	2	2.8
	Secondary	25	34.7
	Tertiary	37	51.4
	Postgraduate	8	11.1
Occupation^+^	Civil servant	22	61.1
	Business/Trader	11	30.6
	Retired	3	8.3
Number of eligible daughter(s)^+^	One	50	69.4
	Two	22	30.6

^+^For only parents

In order to understand the perception of the parents, caregivers and school administrators about HPV and HPV vaccine, they were asked questions about their awareness of the vaccine, reason for accepting the vaccine, perceived severity of HPV, perceived susceptibility of HPV, and thoughts on the relevance of the vaccine. The key findings are summarized in Table 3.

**Table 3:** A thematic matrix of key findings from the study

Main Theme	Sub-themes	Description / Participant Insights
Awareness of Free HPV Vaccine	School-based delivery awareness	Parents noted that vaccines were administered free of charge at their children’s schools.
	Church-based delivery awareness	Some mentioned receiving information or access through churches.
	Cost-related barriers previously	Prior knowledge of the vaccine existed, but financial barriers had prevented access before the free campaign.
	Target group recognition	Correct understanding that the vaccine was intended for girls aged 9–14 years.
Reasons for Vaccine Acceptance	Cancer prevention motivation	Vaccine accepted as a preventive tool against cervical cancer.
	Protective attitude towards children	Desire to give children the best chance for good health.
	Influence of prior awareness or education	Parents who had been previously informed about HPV and the vaccine were more likely to agree to it.
	Health-conscious parenting	Some viewed immunization as part of being a responsible parent.
Perceived Severity of HPV	Health implications	Descriptions of HPV as leading to severe outcomes like cancer, infertility, or death.
	Educational and financial disruption	Fears that HPV infection could disrupt school and incur high treatment costs.
	Long-term impact on girls’ lives	Concerns over HPV affecting future wellbeing and opportunities.
Perceived Susceptibility to HPV	Belief in universal risk	Recognition that HPV risk is not limited to sexually active or older girls—“any girl could get it.”
	Hygiene and environmental factors	Some attributed susceptibility to dirty toilets or poor hygiene.
	Skepticism about personal risk	A few parents doubted their daughters were at risk due to perceived controlled upbringing.
	Societal influences	School administrators and some parents cited early sexual activity, peer influence, or societal shifts as increasing susceptibility.
Perception of HPV Vaccine Relevance	Long-term protection focus	Seen as a valuable preventive health investment with future benefits.
	Desire for broader age group access	Parents expressed regret that older girls (above 14) were not eligible.
	Confidence in the vaccine	Trust was built due to absence of adverse reactions and official endorsement.
	School-level benefit	School administrators highlighted the vaccine’s importance in maintaining school health and minimizing disease spread among students.

### Awareness of free HPV vaccine

Many parents and caregivers demonstrated a clear awareness that the HPV vaccine was being offered free of charge, a factor that appeared to positively influence their acceptance and uptake of the vaccine. Several respondents acknowledged that announcements and community sensitization efforts had explicitly communicated the vaccine’s availability at no cost. Religious and community leaders played a pivotal role in disseminating information, enhancing awareness across broader networks. Others were aware not only of the vaccine’s free status but also of logistical details, such as where it could be accessed and who was eligible. The cost-free nature of the vaccine was especially important for caregivers who had previously faced financial barriers.

*“Yes*,* I know and based on the announcement*,* we were told that it is free and we would not pay money and that was how it was*,* we didn’t pay for it”* **Parent/IDI/Ifelodun LGA/Kwara**.

*“Well*,* here we have a chaplain who is taking care of the school. So*,* when they came for the vaccination*,* the message got to him and when he confirmed about the vaccination*,* he has to announce it in the church so that parents should allow their children to take the vaccination”* **Head Teacher/IDI/Ezza North LGA/Ebonyi**.

*“Yes I was aware that it is free and can be found in some specific location including health facilities. And that the target group is 9–14 years”* **Parent/IDI/Katsina LGA/Katsina**.

*“Then I couldn’t afford it*,* so my first daughter missed it. Because it’s a very huge money. So when I*,* this year was the last year that we*,* which was this year now*,* that we heard of it and got the opportunity of meeting it again free from the government. So I’m very happy it’s indeed a vaccine and a good one for that matter.”* **Parent/IDI/Oshimili South LGA/Delta**.

### Reasons for HPV vaccine acceptance

Parents and caregivers articulated a range of motivations for accepting the HPV vaccine for their daughters, with a strong emphasis on disease prevention, particularly the prevention of cervical cancer. For many, the vaccine represented a proactive measure to safeguard future health. This belief in the protective value of the vaccine was echoed by others who had been influenced by personal experiences and prior knowledge about the risks associated with HPV. In some cases, awareness of the dangers posed by the virus created a sense of urgency and readiness among both parents and children.

*“Number one is to prevent the cancer. That is the primary motive*,* to prevent it not to occur although we did not pray for such. It is good to be on the preventive side. Like the—the saying they always say*,* ‘Prevention is better than cure’.”* **Parent/IDI/Oredo LGA/Edo**.

*“As a parent I saw it as a program that will help us a lot because of several things I have heard and experiences I have heard about the virus*,* so when the opportunity came then I made up my mind to ensure they take it so that the virus will not affect them in the future.”* **Parent/IDI/Offa LGA/Kwara**.

*“Some of them have heard about that vaccine*,* so they are willing to take it because they know the danger of that virus. So*,* they are willing*,* even before telling them*,* some of them will say*,* ‘Ahh*,* we have heard about that vaccine…mummy let them come.’ So*,* they heard about it and so we don’t have any issue in that.”* **Class Teacher/IDI/Ido LGA/Ekiti**.

Participants also reflected on broader values related to health and responsibility. One caregiver emphasized the importance of intentional health-seeking behaviour, illustrating how vaccine acceptance was linked not only to immediate concerns about disease prevention but also to a broader understanding of reproductive health and parental responsibility.

*“Well*,* health is wealth. And if a vaccine is released for your health and safety*,* you have to be intentional enough to make sure you are healthy*,* your children are healthy. Most especially*,* the cervix is a very important organ for every woman. Hence*,* you don’t just ignore it when there are vaccines to make it healthy. So*,* these are some of the factors that influenced my decision.”* **Parent/IDI/Calabar Municipal LGA/Cross River**.

### Perceived severity of HPV infection

#### Parents’ perception of severity of HPV infection

Parents and caregivers generally perceived HPV as a serious health threat, with far-reaching consequences for affected individuals and their families. This perception was often framed in terms of the potential progression of the virus to cervical cancer and the subsequent impact on multiple aspects of a girl’s life. The seriousness of HPV was also linked to its association with cancer, as participants recognized the virus as a precursor to life-threatening conditions. Most strikingly, some parents viewed HPV as ultimately fatal, reflecting a high level of concern about the physical suffering and mortality associated with HPV and suggest that fear of severe outcomes may be a strong motivator for vaccine acceptance.

*“It will affect their family because economically*,* it will affect their lives. They have to take*,* maybe*,* to hospital. And when they take her to hospital*,* they have to spend money. And you can see*,* economically*,* they have affected. It can also affect her educational background. Because when she is ill*,* she can no longer go to school. She cannot continue her education*,* some sort of that nature.”* **Headteacher/IDI/Kurfi LGA/Katsina**.

*“If the person is having the virus there is tendency for that person to have the cancer. Since the virus is already there but to prevent it now is good. Vaccines are given to these female children.”* **Parent/IDI/Oredo LGA/Edo**.

*“It will bring her death. Before death She will go through a lot of pain.”* **Parent/IDI/Bakinladi LGA/Plateau**.

#### School administrators’ perception of severity of HPV infection

School administrators expressed significant concern regarding the potential impact of HPV on students’ health, education, and the broader school environment. The perceived severity of the virus was rooted in its capacity to cause serious illness and even death, thereby disrupting the lives of affected students. Beyond individual health outcomes, school administrators were also concerned about the implications of HPV infections for the school community as a whole. The prospect of viral transmission within school settings was seen as a serious threat, as highlighted by one of the respondents. Furthermore, administrators linked HPV infections to educational disruptions, including absenteeism and school dropout.

*“Well*,* it will affect them because when it affected them*,* they will become sick. And when they become sick*,* you know*,* it has affected their life. Because maybe*,* probably*,* that can lead them to death.”* **Headteacher/IDI/Kurfi LGA/Katsina**.

*“So many ways. Okay. You know school is a group or a group of people coming together. So*,* if anyone or 2 persons is infected with the virus*,* there is a tendency that there will be a widespread in the school.”* **School Administrator/IDI/Bakassi LGA/Cross River**.

*“It will affect the school quite right because several of them will have to*,* they will drop out from school and that is why*,* if we can come up to fight against it immediately*,* it keeps the student.”* **Headteacher/IDI/Ogori-Mangogo LGA/Kogi**.

### Perceived susceptibility of HPV infection

#### Parents’ perceived susceptibility of daughter to HPV infection

Parental perceptions of their daughters’ susceptibility to HPV revealed a complex interplay of awareness, uncertainty, and culturally shaped understandings of risk. While some caregivers acknowledged the general risk of infections, they did not necessarily perceive their own children as vulnerable. One respondent expressed this ambivalence,

*“Humanly speaking*,* I know that anyone can be at risk of several infections but I have never had such a thought towards my daughters. But I don’t know what can happen in the future.”* **Parent/IDI/Offa LGA/Kwara**.

Other parents expressed misconceptions about how HPV is transmitted, yet still supported vaccination as a preventive strategy showing a belief in non-sexual modes of transmission, suggesting gaps in biomedical understanding but simultaneously reinforcing motivation to seek protection through vaccination. Some parents viewed children’s behaviour—especially unsupervised play—as a source of potential risk, prompting precautionary action.

*“Yes and No because when a female child squats to pass urine*,* she can be infected not necessarily sexual intercourse. The hot ground when she bends to urinate can cause it*,* that’s why we said our children should take the vaccine.”* **Parent/IDI/Ido LGA/Ekiti**.

*“You know children of nowadays you do not know how they play. So*,* I said let me bring her to be vaccinated to stop any sickness that is coming.”* **Parent/IDI/Jos South LGA/Plateau**.

#### School administrators’ perceived susceptibility of students to HPV infection

School administrators generally perceived students as vulnerable to HPV infection, particularly in the context of limited awareness and inadequate sexual health education. A recurring theme in their responses was the belief that ignorance—both among students and the broader community—heightens susceptibility. One administrator emphasized this point, highlighting the role of structural and socio-cultural factors, such as early marriage and lack of knowledge, in increasing students’ risk.

*“They are vulnerable because of illiteracy*,* ignorance. But when we enlighten our people to know the danger of this sex early sex*,* you see*,* or*,* I can tell you*,* or even any marriage to marry a small girl*,* without knowing anything about how to prevent herself from contracting diseases*,* and what have you. So this are one of the problems.”* **Education Secretary/IDI/Kurfi LGA/Katsina**.

The absence of adequate health education and awareness campaigns was also seen as a contributing factor to risk. Administrators acknowledged the unpredictability of exposure, while further rejecting the notion that religious or cultural identity conferred protection against HPV.

*“They are at risk if they don’t have the idea of this information*,* when there is no enlightenment by these people that carry out this exercise*,* there is high risk.”* **Principal/IDI/Offa LGA/Kwara**.

*“Because no one can tell*,* so we cannot say that this thing cannot happen to this…because*,* looking at the way the virus is spreading now*,* so there is no one that can say that this thing cannot happen to this err pupil*,* it is only God that can take control. That is why we don’t deny anyone of them*,* especially those of them that are willing to take it.”* **Class Teacher/IDI/Ido LGA/Ekiti**.

*“I believe we have a dynamic society now*,* everything is possible. We cannot say we are Muslims and Christians this cannot happen. Whatever we need to prevent*,* let us prevent it at least it cause nothing not to be cautious.”* **Headteacher/IDI/Okene LGA/Kogi**.

### Perception of the HPV vaccine relevance

Parents, caregivers and school administrators overwhelmingly viewed the HPV vaccine as a relevant and valuable intervention for safeguarding the health of their students. The preventive nature of the vaccine was particularly emphasized, with administrators recognizing its role in protecting against future infection. They also acknowledged the practical difficulties of controlling students’ behaviour and emphasized that vaccination offered a more reliable form of protection. The vaccine was also seen as providing a sense of security and assurance.

*“It is very important because the vaccine the aim is to prevent from such infection. We are even saying if not for the cost the vaccine should have been all round not only for age 9 to 14. It should have been for the whole of the students.”* **Principal/IDI/Jos South LGA/Plateau**.

*“It will prevent them from getting the virus because there is no way you can guide the female student they can be involve in sexual act and since that is the only way it can be transmitted so they be at risk.”* **Headteacher/IDI/Oredo LGA/Edo**.

*“I reason that the students having being vaccinated are secured against the risk*,* the infection.”* **School Administrator/IDI/Bakassi LGA/Cross River**.

In addition to its health benefits, administrators also highlighted the vaccine’s acceptability and the absence of adverse effects as factors that contributed to its perceived value. This positive experience reinforced their support for the intervention and strengthened its relevance in the school health agenda.

*“Basically*,* since it’s something that has to do with health*,* of course*,* we embraced it*,* as usual*,* for the benefit of the children*,* believing that there will not be any side effects*,* so we have not had any and I think we’ve had it for months now*,* we’ve not had any reports. So*,* it’s beautiful.”* **Principal/IDI/Ifelodun LGA/Kwara**.

## Discussion

### Awareness and reasons for HPV vaccine acceptance

This study found that awareness of the HPV vaccine was generally high among parents, caregivers, and school administrators in the sampled regions, particularly about its availability at no cost and its targeting of girls aged 9–14. This awareness was primarily facilitated through school-based campaigns, announcements in religious institutions, and direct communication from school officials. For many parents, the cost-free nature of the vaccine was a critical enabler, especially for those who had previously been unable to afford it. These findings align with research from various Nigerian contexts and other LMICs [20, 21, 31–33]. Knowledge and perceived benefits of the HPV vaccine are significant predictors of acceptance among mothers, particularly when the vaccine was offered free of charge and delivered through familiar institutions like schools and churches [34–36]. A study conducted in Ghana found that parents and girls who had received accurate information through health workers or community leaders were more likely to accept HPV vaccination for their children [37], further stressing the importance of communication and leveraging existing structures for new vaccine introduction.

Acceptance of the vaccine was largely rooted in a strong appreciation for its preventive value. Parents commonly cited the desire to protect their daughters from future health risks, especially cervical cancer, as a motivating factor. For some, previous exposure to stories or lived experiences related to cancer reinforced the importance of prevention. Additionally, the perception that the vaccine was endorsed by trusted institutions—such as schools, churches, and the government—bolstered confidence and willingness to participate in the vaccination exercise. Studies have shown that HPV and HPV vaccine awareness and acceptance are not uniformly high across Nigeria [20, 21, 23, 25]. Research from northern regions has consistently shown lower awareness and uptake due to factors such as limited access to information, cultural barriers, and vaccine hesitancy rooted in distrust of government programs [38]. This regional disparity underscores the importance of context-specific strategies in promoting HPV vaccination [39].

### Perceived severity, susceptibility, and relevance of the HPV vaccine

Participants demonstrated a high level of concern about the potential severity of HPV infection, particularly its link to cervical cancer, physical suffering, and even death. Parents and school administrators alike articulated the belief that HPV-related illness could disrupt girls’ education, diminish their quality of life, and cause significant emotional and financial hardship for families. This perception and the protective benefits of vaccination are well documented in literature from both high- and low-income countries [34, 40–43]. Studies from Kenya [44], Tanzania [45], and Uganda [46] have reported that knowledge of the link between HPV and cervical cancer is a strong driver of vaccine uptake. However, like in the current study, these perceptions are often accompanied by misconceptions about HPV transmission, especially in communities with limited sexual health education [47].

In terms of susceptibility, parents expressed mixed beliefs. Some believed that HPV infection could occur through non-sexual means such as poor hygiene or environmental exposure like urinating on hot ground, indicating misconceptions about the virus’s transmission. Others, however, recognized the role of risky behaviours and early sexual activity in increasing vulnerability. School administrators also perceived students as susceptible, citing factors such as low awareness, peer influence, and the inability of parents and schools to monitor all aspects of students’ behaviour. In a systematic review and meta-analysis conducted about HPV vaccination uptake in sub-Saharan Africa, Asgedom et al. [48]. observed that while many parents supported vaccination due to its protective function, others remained unsure about their children’s risk, sometimes underestimating susceptibility or misunderstanding how HPV is transmitted. These misconceptions can hinder informed decision-making and must be addressed through comprehensive, age-appropriate education initiatives [49].

Importantly, both parents and school personnel overwhelmingly regarded the vaccine as a relevant and beneficial health intervention. The perception that the vaccine provides long-term protection and comes with no side effects reinforced the belief that it should be embraced more broadly, even beyond the current target age group. Additionally, a dissonance emerged between perceived severity and perceived susceptibility to HPV infection. While respondents acknowledged the seriousness of HPV, many did not consider themselves or their children at risk, highlighting a critical gap that could be addressed through targeted behavioural interventions.

## Policy and practice implications

1. Utilizing Trusted Community Structures for Vaccine Delivery.

The findings of this study highlight the effectiveness of school-based delivery platforms and religious institutions in disseminating vaccine information and building trust. Policymakers and immunization program planners should leverage these existing structures to broaden the reach of HPV vaccine campaigns in the future. Schools, in particular, should be maintained as trusted entry points for vaccine delivery and broader health promotion.

2. Strengthening Communication and Community Engagement.

In order to address lingering scepticism, consistent and culturally sensitive messaging—delivered in local languages and endorsed by influential community figures—can further normalize the HPV vaccine. To improve national coverage, targeted interventions should focus on areas with historically low vaccine awareness and uptake. These should include community mobilization efforts, integration of HPV vaccine education into school curricula, and partnerships with religious and traditional leaders. These efforts can help ensure broader acceptance and understanding among parents, teachers, and community leaders.

3. *Advancing Policy*,* Logistics*,* and Long-Term Planning*.

Sustained advocacy for routine inclusion of the vaccine in national immunization programs, coupled with robust logistics support to ensure vaccine availability, will be critical for long-term success. The existing belief in the vaccine’s relevance provides a strong foundation for promoting continuous widespread HPV vaccination. However, this potential will only be realized if misconceptions about HPV transmission are directly addressed. Public health campaigns must include clear, culturally appropriate explanations of how HPV is spread and why girls aged 9–14 are targeted. This will help counter harmful myths and build a scientifically accurate understanding among parents, teachers, and community leaders.

4. *Expanding Access and Promoting Adolescent Health Literacy*.

Support from parents and school officials for extending the vaccine to other age groups suggests an unmet demand. While prioritizing 9–14-year-old girls aligns with WHO recommendations, long-term planning should consider catch-up vaccination strategies for older adolescents who may have missed the opportunity. Building the capacity of teachers and school health personnel to act as health educators could have a multiplier effect, promoting not just HPV vaccine uptake but broader adolescent and reproductive health literacy. Furthermore, integrating HPV vaccine discussions into broader sexual and reproductive health education will equip young people with the tools to make informed decisions about their health.

### Strengths and limitations

One of the strengths of this study lies in its qualitative design, which enabled a nuanced exploration of the beliefs, motivations, and contextual factors influencing HPV vaccine perceptions among parents, caregivers, and school administrators. By incorporating voices from different stakeholder groups, the study captures a more holistic view of the sociocultural and institutional influences on HPV vaccine acceptance. The use of direct quotes also grounds the findings in lived experiences, offering rich, contextual insight that can inform future interventions. The inclusion of participants from rural, urban and peri-urban settings further strengthens the study’s relevance, allowing for a broader understanding of how awareness and acceptance vary across different socioeconomic contexts.

However, the study also has several limitations. Firstly, findings may not be generalizable beyond the specific locations sampled, as is common with qualitative studies utilizing purposive sampling. Secondly, social desirability bias may have influenced participants’ responses, especially in school settings where administrators may feel compelled to support government-led health initiatives. Thirdly, while the study explored perceptions, it did not include girls themselves in the interviews — the primary beneficiaries of the vaccine — whose perspectives would provide additional insight into acceptability and decision-making processes.

Future research should explore the perspectives of adolescent girls and incorporate longitudinal designs to assess how perceptions evolve over time, particularly as HPV vaccine programs become more institutionalized and routinized.

## Conclusion

In conclusion, this study highlights that among parents and school administrators in Nigeria, there is a high level of awareness of the HPV vaccine, particularly as it was provided free of charge through different strategies, including school-based campaigns. Acceptance of the vaccine is largely driven by an understanding of its preventive benefits and a desire to protect girls from the long-term consequences of HPV infection, including cervical cancer. However, the findings also reveal persistent misconceptions about transmission pathways and mixed beliefs about susceptibility, which could limit informed uptake. School administrators were found to be key facilitators in the vaccine communication process and generally expressed strong support for the vaccine’s relevance. Their role—as trusted messengers and health gatekeepers—can be leveraged more effectively in national and local HPV vaccine delivery strategies.

To sustain and expand HPV vaccine coverage, national immunization programs must invest in culturally tailored education campaigns that address both knowledge gaps and misconceptions. The integration of HPV vaccine promotion into broader adolescent health education, especially within school curricula, will be essential. As Nigeria continues to scale up its HPV vaccination efforts, ensuring equitable access, enhancing stakeholder engagement, and addressing sociocultural concerns will be critical to maximizing vaccine uptake and reducing the burden of cervical cancer in the long term.

## Supplementary Information

Below is the link to the electronic supplementary material.

Supplementary Material 1

Supplementary Material 2

## Data Availability

Data is provided within the manuscript.

## Code availability

Yes.
