# Supplementary material for: Perceptions of HPV and HPV vaccines among parents and caregivers of girls aged 9–14 years in Nigeria: a qualitative study
Source: Discov Public Health. 2025 Dec 21;22(1):856. doi: 10.1186/s12982-025-01276-0 (PMC12719340; doi:10.1186/s12982-025-01276-0)
Supplement: Supplementary file 1 — Supplementary Material 1 [file 12982_2025_1276_MOESM1_ESM.docx]

**In-Depth Interview Guide for Parents**

IDI No.: ________

**Informed Consent (Oral)**

Good day Sir/Ma, my name is _____________________ and I work for Sydani Group. My organization is currently undertaking a study titled “**School and Community-based Adolescent Vaccination: Enablers and Barriers from HPV Vaccine Introduction in Nine (9) Nigerian States”**. The study seeks to document the barriers and facilitators of the HPV vaccine introduction and uptake in your state. I would appreciate it if you could spare some of your time to answer some questions. I assure you that all information shared with me shall be kept in utmost confidentiality. Although the interview is voluntary and you have permission to exit at any time, I would appreciate it if you could complete the interview. Please note that this interview session will be recorded to document what is being discussed adequately.

Do I have your permission to go ahead with the interview? Yes/No

*(End the interview if no, and continue if yes)*

**SECTION A: Socio-demographics**

1. Please, introduce yourself.

Focus: *Prompt where the participant skips any of the following*

- Gender
- Level of educational attainments
- Age
- Occupation
- Did you have an eligible daughter in school during the vaccination exercise? (**Probe**: Boarding student or day student)

**SECTION B: Awareness of HPV and the HPV vaccine**

- What have you heard about the HPV vaccine and what do you think about the vaccine?
- Please describe how/where you first heard about the vaccine.

**SECTION C: Perceived susceptibility, severity and benefits of the vaccine**

- Before the vaccination, did it ever occur to that your daughter was susceptible to HPV? And why?
- What do you think are the consequences for anyone infected with HPV?
- Were you aware that the vaccine was free and would be available in your community/health facilities and other locations across?
- What influenced your decision to vaccinate your daughter?
- What are your recommendations on how to improve adolescent vaccination?

*Thank you for your time.*
