# Supplementary material for: Perceptions of HPV and HPV vaccines among parents and caregivers of girls aged 9–14 years in Nigeria: a qualitative study
Source: Discov Public Health. 2025 Dec 21;22(1):856. doi: 10.1186/s12982-025-01276-0 (PMC12719340; doi:10.1186/s12982-025-01276-0)
Supplement: Supplementary file 2 — Supplementary Material 2 [file 12982_2025_1276_MOESM2_ESM.docx]

**In-Depth Interview Guide for School Administrators**

IDI No.: ________

**Informed Consent (Oral)**

Good day Sir/Ma, my name is _____________________ and I work for Sydani Group. My organization is currently undertaking a study titled “**School and Community-based Adolescent Vaccination: Enablers and Barriers from HPV Vaccine Introduction in Nine (9) Nigerian States”**. The study seeks to document the barriers and facilitators of the HPV vaccine introduction and uptake in your state. I would appreciate it if you could spare some of your time to answer some questions. I assure you that all information shared with me shall be kept in utmost confidentiality. Although the interview is voluntary and you have permission to exit at any time, I would appreciate it if you could complete the interview. Please note that this interview session will be recorded to document what is being discussed adequately.

Do I have your permission to go ahead with the interview? Yes/No

*(End the interview if no, and continue if yes)*

**SECTION A: Socio-demographics**

1. Please, introduce yourself.

Focus: *Prompt where the participant skips any of the following*

- Gender
- Level of educational attainments
- Age
- Designation
- Number of years serving in that designation
- Number of years working in the school

**SECTION B: Knowledge and awareness of HPV and the vaccine**

- What do you understand about HPV and how it is transmitted?
- What are the diseases caused by HPV infection?
- Have you heard about the HPV vaccines and what it is used for?
- What age group of girls were eligible for the vaccination?
- Can you describe your involvement in the HPV vaccination program at your school?

**SECTION C: Perceived factors that influenced the vaccine introduction & uptake**

**Perception**

- What are your thoughts about the female students’ susceptibility to HPV infection? (**Prompt:** do you think they may be at risk to the virus based on their health behaviours)
- In what ways do you think the students and/or the school will be affected should they contract the virus?
- What are your thoughts on the relevance of the vaccine to your female students?

**Probe**: how did this affect your attitude towards the vaccination program in your school?

**Education and Communication**

- What were the approaches you employed to communicating the HPV vaccination program to students and parents?
- What were the reactions towards the vaccine and the vaccination program from parents and students?
- What do you think influenced parents’ and students’ participation in the sensitization session?

*Thank you for your time.*
